# Supplementary material for: Predicting visual function by interpreting a neuronal wiring diagram
Source: Nature. 2024 Oct 2;634(8032):113–23. doi: 10.1038/s41586-024-07953-5 (PMC11446822; doi:10.1038/s41586-024-07953-5)
Supplement: Supplementary file 6 — CRF and ERF predictions for individual TmY4 and TmY9 cells. Analogous to Supplementary Data 3, but for TmY target types. Shown are the top four monosynaptic pathways, the strongest pathway passing through each of the top ten intermediary types (ranking from Extended Data Fig. 7), and the trisynaptic pathway Tm1–TmY–Dm3–TmY (see the section entitled Prediction of spatial normalization). [file 41586_2024_7953_MOESM6_ESM.zip › DataS4/TmY9qΓèÑ/720575940627869461.pdf]

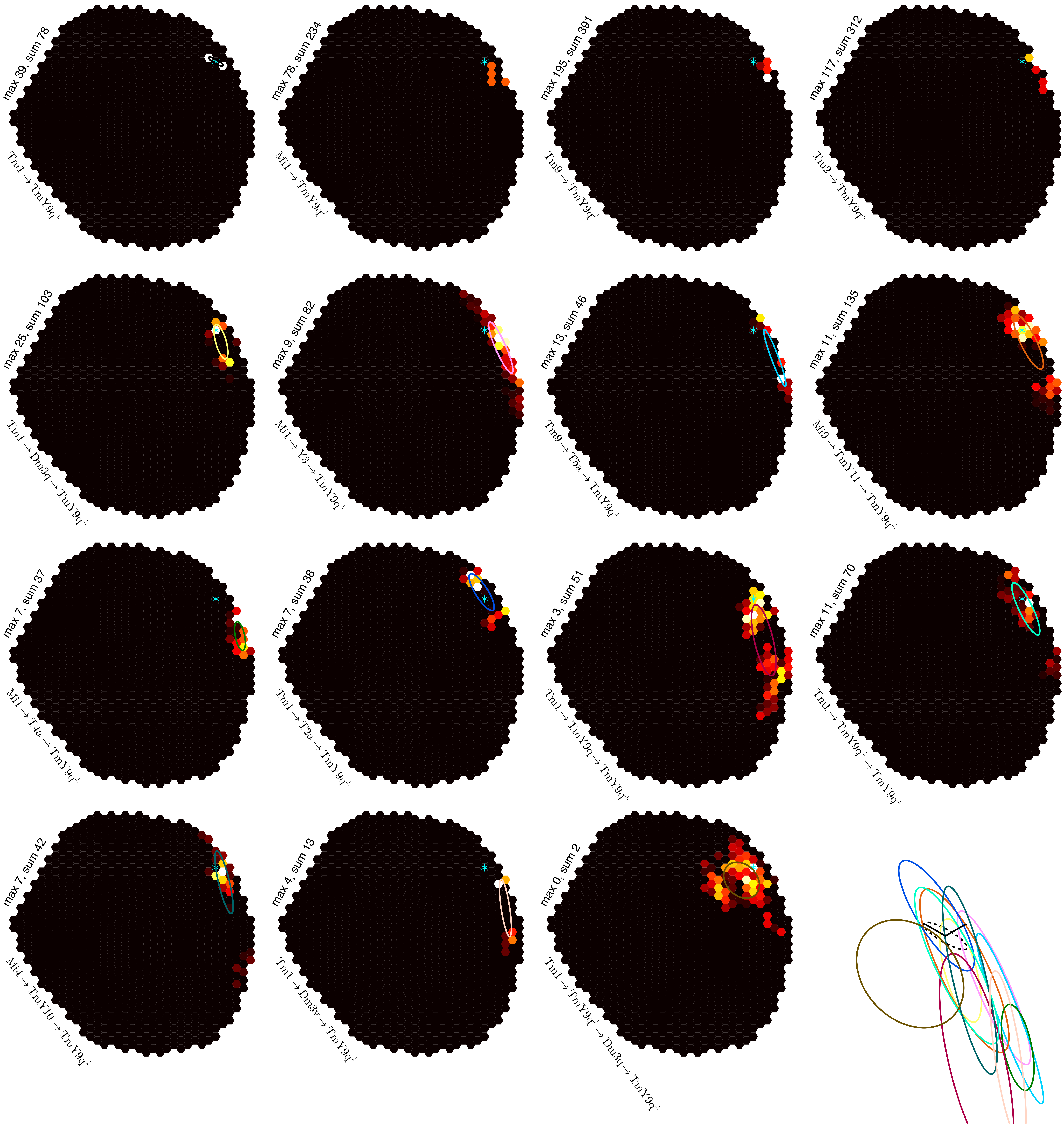
max 39, sum 78
Tm1 → TmY9q⊥
max 78, sum 234
Mi1 → TmY9q⊥
max 195, sum 391
Tm9 → TmY9q⊥
max 117, sum 312
Tm2 → TmY9q⊥
max 25, sum 103
Tm1 → Dm3q → TmY9q⊥
max 9, sum 82
Mi1 → Y3 → TmY9q⊥
max 13, sum 46
Tm9 → T5a → TmY9q⊥
max 11, sum 135
Mi9 → TmY11 → TmY9q⊥
max 7, sum 37
Mi1 → T4a → TmY9q⊥
max 7, sum 38
Tm1 → T2a → TmY9q⊥
max 3, sum 51
Tm1 → TmY9q → TmY9q⊥
max 11, sum 70
Tm1 → TmY9q⊥ → TmY9q⊥
max 7, sum 42
Mi4 → TmY10 → TmY9q⊥
max 4, sum 13
Tm1 → Dm3v → TmY9q⊥
max 0, sum 2
Tm1 → TmY9q⊥ → Dm3q → TmY9q⊥
